# Differences in Referral Access to Care Between Gastrointestinal Subspecialty Patients: Barriers and Opportunities

**DOI:** 10.1089/heq.2018.0001

**Published:** 2018-06-01

**Authors:** Kartika Reddy, Caitlyn Patrick, Hammad Liaquat, Edmundo Rodriquez, Abigail Stocker, Barbra Cave, Matt C. Cave, Laura Smart, Teresa Cutts, Thomas Abell

**Affiliations:** ^1^ Department of Medicine, University of Louisville, Louisville, Kentucky.; ^2^ University of Louisville Hepatitis C Center, Louisville, Kentucky.; ^3^ Department of Social Sciences and Health Policy, Wake Forest School of Medicine, Winston-Salem, North Carolina.

**Keywords:** access to care, gastrointestinal motility disorders, hepatology, liver diseases

## Abstract

**Purpose:** Referral access to subspecialty care for patients with gastrointestinal (GI) diseases is not well defined, but has significant importance to patients. We hypothesized that patients experience barriers to care in two common gastroenterology subspecialties, Hepatology and Motility, in a university medical center.

**Methods:** Two hundred thirteen clinic patients (mean age 46.5 years; 66.5% female; 85.6% Caucasians) completed a formatted questionnaire on access to care. Hepatology patients were older (49.7 years, *p*=0.008); motility patients predominantly female (76.8%, *p*<0.001). Gender distribution was even for hepatology (51.2% female). Both groups were overweight (mean body mass index 28.4).

**Results:** Patients waited a mean 89.5 days to be seen by a subspecialist. There were differences by subspecialty (107.6 days for motility vs. 64.3 days for hepatology, *p*=0.022). A larger percentage of motility patients were told nothing was wrong with them (16.8%, *p*<0.01) and could not be helped (42.1%, *p*=0.000).

**Conclusions:** Access to care for subspecialty gastroenterology patients in a university center appears to be impacted by a number of variables. While there are similarities, differences exist between these two subspecialties. Motility patients were more likely to have been told they have nothing wrong with them, suffer setbacks financially, and suffer mood problems. Their wait time for appointments was also greater than hepatology patients. Further investigations of referral access for gastroenterology patients may yield additional insights into disease-specific barriers to accessing subspecialty care.

## Introduction

Liver diseases and gastrointestinal (GI) motility disorders are growing medical problems in the United States with an associated increased demand for treatment of these diseases.^1,2^ Chronic gastrointestinal disorders including gastroparesis (diagnosed by delayed emptying from the stomach) and cirrhosis often require complex, specialized care. Access to subspecialty care for hepatology (liver) patients has received some limited attention in studies; however, access to care is not well defined for either motility or hepatology patients.^3–5^ Most importantly, it has been shown that access to subspecialty care improved 5-year survival of hepatology patients.^6^ Additionally, delays in hospital care have been shown to lead to increased hospital stays and worsened outcomes.^7–11^ Little data exist on the emotional and financial strains caused by the complex GI diseases associated with patients in hepatology and motility subspecialty clinics.^12^ The purpose of this study was to assess the differences in access to care between two GI subspecialties, hepatology and motility, in an academic medical center setting. We aimed to better understand what barriers delay or otherwise impair patients from being seen and treated by subspecialists.

## Methods

### Outcomes

Primary outcomes included differences between wait times for appointments at GI subspecialties, patients' moods and financial difficulties, frequency of hospitalizations for illness, and ease of access to caregivers.

### Patient survey questionnaire

An institutional review board-approved questionnaire was used for the study. All patients referred to clinics with liver or motility diagnoses were asked to complete the questionnaire at the time of their first clinic appointment with the GI provider. Informed consent was obtained from all patients before they completed the questionnaire. The questionnaire contained questions on patient demographics, specific diagnosis, knowledge about their diagnosis and duration of disease, wait time for appointment, previous response by other physicians about their disease, patient's mood, financial difficulties due to illness, frequency of hospitalizations for illness, and ease of access to caregivers. The questionnaire was constructed by two of the authors who have had extensive experience in both patient focus groups and patient reported outcomes (PRO), including development and validation of PRO scales.^13^ Any and all patient identifying information was removed before processing the data. A copy of the questionnaire can be found in Appendix 1.

### Study patients

Subjects included all patients who had been referred to the subspecialty clinic by a primary care provider, due to symptoms meeting clinical guidelines for either hepatology or motility disorders. Two hundred seventy-five consecutive patients were offered the questionnaire and 213 consecutive patients completed it, with an estimated 30% of total number of patients who came through the clinic during the study period declining to participate. Among all participants, the mean age was 46.5 years, 66.5% were female, and 85.6% Caucasian. Hepatology patients were older (49.7 years, *p*=0.008); motility patients were predominantly female (76.8%, *p*<0.001), while gender distribution was even for hepatology participants (51.2% female). There were more African American patients in the hepatology group (20.5% vs. 5% in motility, *p*<0.001). Both groups were overweight (mean body mass index [BMI] 28.4) (Table 1).

**Table 1.** **Patient Characteristics**

Demographic	Combined patient data (*n*)	Motility diagnosis (*n*)	Hepatology diagnosis (*n*)	*p*-value (motility vs. hepatology)
Age (mean with SD in years)	46.5±14.7 (206)	44.3±15 (122)	49.7±13.8 (84)	0.008
BMI (mean with SD in Kg/m^2^)	28.4±9.1 (196)	28.2±10.4 (119)	28.8±6.7 (77)	0.614
Duration of GI condition (mean with SD in months)	72.78±77.2 (180)	76.3±68.2 (120)	65.7±92.9 (60)	0.433
Gender				
Male (%)	33.5 (70)	23.2 (29)	48.8 (41)	<0.001
Female (%)	66.5 (139)	76.8 (96)	51.2 (43)	<0.001
Ethnicity				
Caucasian (%)	85.6 (173)	91.5 (109)	77.1 (64)	<0.01
African American (%)	11.4 (23)	5.0 (6)	20.5 (17)	<0.001
Native American (%)	0.5 (1)	0.8 (1)	0 (0)	<0.4150
Other (%)	2.5 (5)	2.5 (3)	2.4 (2)	<0.9640

*n*=total number respondents to a specific question or variable.

BMI, body mass index; GI, gastrointestinal.

### Statistical methods

Data collected were reviewed for completeness and tabulated even if not all questions were answered by each participant. The number of respondents (*n*) was reported for each question/variable. Descriptive statistics, reported as mean and percentage for each subspecialty and questionnaire item were compared by t-test where statistically possible. Statistical significance was defined as <0.05.

## Results

Motility Patients: The majority of motility patients suffered from gastroparesis (51.6%). The patient wait time for an appointment was on average 107 days. 42.1% were told they could not be helped; 7.9% previously had GI physicians decline to see them; 16.8% were told there was nothing wrong with them; 35.4% had lost their job due to their illness; and 54.8% had suffered financial losses due to their illness. Additionally, 81.1% had suffered mood or social problems. On average, motility patients felt they received inadequate care 2.4 times over the previous month. They reported an average of 3 emergency room (ER) visits over the last year, and they were hospitalized for an average of 7.4 days (Table 3).

Hepatology Patients: The majority of hepatology patients suffered from liver disease secondary to hepatitis C infection (44.7%). The second most common diagnosis was cirrhosis (12.9%) (Table 2). On average, patients with liver disease waited 64 days for an appointment. 5.9% reported that GI physicians had previously declined to see them. About 4.8% of the patients were told that there was nothing wrong with them, and 3.5% of patients were told that they could not be helped. About 10.6% had lost jobs due to their illness, and 20.2% had suffered financial losses due to their illness. About 41.2% of the hepatology patients had suffered mood or social problems due to their illness. Hepatology patients rated their primary care physicians and their gastroenterologists as the providers who were easiest to access. On average, hepatology patients reported 1.4 ER visits over the last year, and required about 2.5 days of hospitalization. Hepatology patients felt that they had received inadequate care 0.5 times over the last month (Table 3).

**Table 2.** **Diagnoses**

Motility	*n* (%)	Hepatology	*n* (%)
Gastroparesis	66 (51.6)	Hepatitis C	38 (44.7)
Vomiting	3 (2.3)	Hepatic steatosis	3 (3.5)
Abdominal pain	6 (4.7)	Cirrhosis	11 (12.9)
IBS	5 (3.9)	Liver disease	3 (3.5)
Dysphagia	4 (3.1)	Autoimmune	1 (1.2)
GERD	4 (3.1)	NASH	1 (1.2)
Constipation	5 (3.9)	Liver nodule	1 (1.2)
Achalasia	1 (0.8)	Jaundice	1 (1.2)
Ischemic colitis	1 (0.8)	FNH	1 (1.2)
Cyclical vomiting syndrome	1 (0.8)		
Chronic pancreatitis	1 (0.8)		
Bloating	1 (0.8)		
Crohn's disease	1 (0.8)		
Barrett's esophagus	1 (0.8)		
Diarrhea	3 (2.3)		
None	21 (16.4)		25 (29.4)
Total	128		85

*n*=total number of respondents to a question or variable.

FNH, focal nodular hyperplasia; GERD, gastroesophageal reflux disease; IBS, irritable bowel syndrome; NASH, nonalcoholic steatohepatitis.

**Table 3.** **Patient Responses**

Questions	Combined patient data (*n*)	Motility diagnosis (*n*)	Hepatology diagnosis (*n*)	*p*-value (motility vs. hepatology)
Patient wait time from time of initial call to appointment (mean with SD in days)	89.5±139.3 (172)	107.61±174.6 (100)	64.3±55.6 (72)	0.022
Patient was told nothing was wrong with them (% yes)	12 (209)	16.8 (125)	4.8 (84)	<0.01
Patient was told that they cannot be helped (% yes)	26.5 (211)	42.1 (126)	3.5 (85)	0.000
No. of physicians that have told them that they cannot be helped (mean with SD)	2.57±2.2 (207)	2.6±2.2 (123)	1.0±1.0 (84)	<0.001
Have other GI specialists declined to see you (% yes)	7.1 (211)	7.9 (126)	5.9 (85)	0.5795
Lost job due to illness (% yes)	25.6 (212)	35.4 (127)	10.6 (85)	<0.001
Suffered financial losses due to illness (% yes)	40.3 (208)	54.8 (124)	20.2 (84)	0.000
Suffered mood or social problems due to illness (% yes)	65.4 (212)	81.1 (127)	41.2 (85)	0.000
Depression (%)	38.9	48.8	23.5	<0.001
Irritability (%)	29.9	37	18.8	<0.001
Anxiety (%)	35.1	44.9	20	<0.001
Social engagements cancelled (%)	32.7	47.2	10.6	0.000
Patient told by PCP that they cannot handle the GI problems anymore (% yes)	76.8 (208)	82.5 (126)	70.7 (82)	0.0465
Which provider saw patient the quickest (1 easiest; 4 hardest in mean with SD				
PCP	1.8±1.0 (141)	1.8±1.0 (95)	1.6±1.0 (46)	0.186
GI doctor	2.3±1.2 (141)	2.5±1.2 (96)	1.8±1.1 (45)	0.001
ER	1.9±1.0 (113)	1.8±1.0 (78)	2.2±1.1 (35)	0.051
Urgent care	2.7±1.1 (77)	2.7±1.1 (54)	2.6±1.2 (23)	0.694
Number of times in past month that patient did not feel they received adequate care (mean with SD)	1.7±3.3 (194)	2.4±4.0 (114)	0.5±1.3 (80)	<0.001
Number of ER visits over last year (mean with SD)	2.3±3.8 (206)	3.0±4.5 (123)	1.4±2.0 (83)	0.001
Number of overnight hospital stays in the past 1 year (mean with SD)	1.4±3.0 (201)	1.8±3.4 (118)	0.9±2.0 (83)	0.013
Number of days in the hospital over the past 1 year (mean with SD)	5.4±18.4 (202)	7.4±23.5 (118)	2.5±5.5 (84)	0.030

*n*=total number of respondents to a question or variable.

ER, emergency room; PCP, primary care physician.

## Discussion

Referral access to subspecialty care for patients with GI diseases is not well delineated, but has significant importance to patients. Access for ongoing care of chronic medical problems has been shown to decrease morbidity and mortality.^5^ In this study, we aimed to study and compare access to care to two GI subspecialities, hepatology and motility, in a university medical center setting. Two hundred thirteen consecutive patients were evaluated by a formatted questionnaire on access to care.

While there are many similarities, differences exist between the subspecialties of hepatology and motility. The motility patients were on average slightly younger than the hepatology patients. The motility group had a greater percentage of female patients, while the hepatology group was more equally distributed between male and female. The hepatology group had a larger percentage of African American patients. Motility patients had longer wait times for an appointment and were more likely to be told there was nothing wrong with them or that they could not be helped. Motility patients were also more likely to suffer financial losses, job losses, and mood or social problems due to their illness and were more likely to feel they had received inadequate care over the last month. The motility patients also required more ER visits over the last year in comparison to the liver patients. Both groups of patients had lengthy wait times of over 2 months, though motility patients usually waited over 3 months for subspecialty care.

Access to care for subspecialty GI patients in a university medical center appears to be impacted by a number of variables. While patient access to care is likely due to a complex set of issues, this pilot study highlights some of those issues, in terms of the patient's perceptions of care, as reported via a structured questionnaire. Access to care is a significant medical problem^2,7^ and access to subspecialty care for hepatology patients has been studied only with patients within the Veterans Affairs healthcare system.^6^ We found no other studies that examined access to subspecialty care for motility patients. This report is the first study that we are aware of that examined access to subspecialty care comparing hepatology and motility patients, and it highlights the disparity in access to care for motility and hepatology patients. Given that patients were told that there was nothing wrong with them, or that they could not be helped, one future direction to improve access to subspecialty care could be educating referring providers on the disease process, interventions offered by subspecialists, and how referral could improve patient conditions. Additionally, use of volunteer peers who have like conditions could be provided to help both motility and hepatology patients navigate the healthcare system in a more efficient manner and/or aid them in finding and utilizing other resources for care. Further investigations of referral access for GI patients may yield additional insights into disease-specific barriers to accessing subspecialty care that will help inform future possible interventions to improve access.

### Health equity implications

This pilot study demonstrates the difficulties that patients suffering from gastrointestinal illnesses with specialized needs may have in obtaining appropriate care. Larger studies of access to care with other GI diseases, particularly cross-site comparisons to improve generalizability of such findings, may be needed to fully evaluate barriers to care, and then address them.

### Limitations

This study is based on a patient-completed questionnaire; therefore results depended on patient participation and accurate responses to questions. Roughly, 30% of those offered the questionnaire declined participation, so persons who self-selected to participate may have different characteristics than those who did participate. For example, those who opted to take the questionnaire might have been experiencing less acute symptoms or distress than those who declined. The questionnaire was not validated before this study; however, it was developed for this specific inquiry as no previously validated questionnaires were available. This study took place during the time when our medical center was undergoing a number of changes in referral patterns, due in part to increased number of persons seeking care, as Medicaid coverage for more under-served persons expanded at state level. These changes could have affected the patients seeking access to care. Lastly, both the motility clinic and hepatitis C clinics were relatively new to our medical center, which also may have affected patient referral patterns.

## Conclusions

Access to care for two distinct GI subspecialties in an academic medical center revealed both similarities and differences. This pilot study demonstrated the difficulties patients suffering from gastrointestinal illnesses with specialized needs may have in receiving care. Larger studies on access to care with other GI diseases may be needed to fully evaluate barriers to care, and then address them.

## Ethics

This study was approved by the University of Louisville's Institutional Review Board, under reference number 14.0101.

## Abbreviations Used

BMI: body mass index
ER: emergency room
GI: gastrointestinal
PRO: patient reported outcomes
PCP: primary care physician

## Appendix 1: Patient survey

Please complete and return to receptionist

Diagnosis or reason for appointment:

Age:  Race:  Hispanic:  Y___ N___  Gender: M___ F___

Weight: ____lb  Height___ft ___in

 1. How long have you been suffering with a chronic GI condition? _______months _______years
 2. What condition is it? _______________________________________________________________
 3. How long have you been waiting to be seen in our clinic, since you first called for an appt? _______
 4. Have you been told there is nothing wrong with you? _________yes _________no
 5. Have you been told that you cannot be helped? _________yes _________no
 6. If so, how many doctors? ____________
 7. Have other GI specialists declined to see you due to your illness? _________yes _________no
 8. Have you lost a job related to illness? __________yes __________no
 9. Have you suffered financial setbacks related to illness or its care? _________yes _________no
10. Have you suffered social or mood problems related to illness, can you specify? (for example, depression, irritability, anxiety, canceling social engagements) ________yes ________no
   If yes: ______________________________________________________________________
11. Has your existing primary care physician shared with you that he/she cannot handle your GI problems and that you have to see a GI specialist? __________yes __________no
12. Of the providers listed below, who sees you the quickest for your GI symptoms? (rank on a scale of 1 (easiest) to 4 (hardest))?
_____ Primary care physician
_____ GI doctor
_____ Emergency Room
_____ Minor Medical Center or Urgent Care
13. How many times within the past 12 months have you felt that you could not receive the care you needed? __________
14. How many ER visits have you had in the past 12 months? ________________
15. How many overnight hospitalizations have you had in the past 12 months, and how many days did you spend in the hospital?
___________________visits, ______________________ number of days in hospital
